# Redirecting engineered immune cells using G protein-coupled receptors in cancer therapy

**DOI:** 10.1016/j.iotech.2026.101582

**Published:** 2026-01-10

**Authors:** W. den Hartog, J. Harwood, S. Kobold

**Affiliations:** 1 Institute of Clinical Pharmacology, LMU University Hospital, LMU Munich, Munich, Germany; 2 German Cancer Consortium (DKTK), Partner Site Munich, a partnership between the DKFZ Heidelberg and the LMU University Hospital, LMU Munich, Munich, Germany; 3 German Center for Lung Research (DZL), Partner Site Munich, Munich, Germany; 4 Einheit für Klinische Pharmakologie (EKLiP), Helmholtz Zentrum München - German Research Center for Environmental Health, Neuherberg, Germany

**Keywords:** GPCR engineering, adoptive cell therapy, cell trafficking, solid tumors, chemokine receptors

## Abstract

Chimeric antigen receptor (CAR) cellular therapy, particularly CAR-T cells, has revolutionized the treatment of hematologic malignancies. However, these therapies show limited efficacy against solid tumors, in part due to the inefficient trafficking of effector cells to the tumor. This review explores the potential of engineering natural and synthetic G protein-coupled receptors (GPCRs) to overcome this migratory hurdle. Chemokine receptors have been the most used GPCR family in this setting. Engineering effector immune cells to express chemokine receptors that match tumor-derived chemokines has been shown to increase their chemotaxis and to improve antitumor efficacy in preclinical models. In addition to improved migration, chemokine receptor engineering can also have additional benefits, such as remodeling of the tumor microenvironment and metabolic rewiring of engineered cells. However, the effectiveness of this approach is limited by the tumor-specific and heterogeneous chemokine milieu. Emerging strategies make use of synthetic GPCRs and could overcome some of these limitations using chemogenetic and optogenetic approaches. Here, mutated GPCRs binding only to specific and orthogonal ligands or light-sensitive channels are used for cell modulation and trafficking. Equipping cells with these synthetic GPCRs allows for precise and stimulus-controlled immune cell migration. Together, natural and synthetic GPCR engineering form promising approaches to enhance immune cell trafficking, persistence, and efficacy.

## Highlights

• GPCRs are key regulators of immune cell migration and form interesting targets for engineering in cancer immunotherapy.
• GPCR engineering via chemokine receptors enhances effector cell migration and infiltration in preclinical solid tumor models.
• Chemokine receptor engineering can enhance metabolic fitness and induce TME remodeling.
• Synthetic GPCR engineering in opto- and chemogenetics enables precise ligand- or light-controlled immune cell migration.
• Natural and synthetic GPCR engineering are promising approaches to enhance CAR cellular therapy efficacy in solid tumors.

## Introduction

The landscape of cancer treatment has been transformed by immunotherapy, offering durable clinical benefits in cases previously resistant to chemotherapy, radiotherapy, or targeted agents.^1^ Its rationale lies in harnessing the patient’s immune system to recognize and eliminate malignant cells.^2^ Among the most successful immunotherapeutic strategies are chimeric antigen receptor (CAR)-T cells. These are autologous T lymphocytes engineered to express synthetic receptors that directly target tumor-associated antigens independently of the major histocompatibility complex. This results in robust antitumor activity. Structurally, CARs are modular synthetic proteins composed of four principal elements: (i) an extracellular single-chain variable fragment (scFv) that recognizes tumor-associated antigens, (ii) a hinge and transmembrane region that anchor the receptor, (iii) an intracellular CD3ζ signaling domain that initiates T cell activation, and (iv) one or more co-stimulatory domains, most commonly CD28 or CD137. These co-stimulatory domains promote proliferation, survival, and persistence.^3^

Seven CAR-T cell therapies have been approved by the United States Food and Drug Administration, all targeting clusters of differentiation (CD) 19 or B-cell maturation antigen (BCMA). These therapies have significantly improved outcomes for patients with advanced and refractory hematologic malignancies such as acute lymphoblastic leukemia, diffuse large B-cell lymphoma, multiple myeloma, and mantle cell lymphoma. This progress has established CAR-T cell therapy as a standard of care in several of these diseases. The translation of CAR-T cells to solid tumors has proven more challenging, although recent clinical trials have shown promising results in brain, liver, and neuroblastoma patients.^4^ Beyond T cells, other immune cell types are also being developed for adoptive cell therapy, with natural killer (NK) cells as the most advanced alternative.^5^

Despite these advances, the efficacy of CAR-T cell therapy is hindered by several cell-intrinsic and extrinsic factors. Intrinsic factors include CAR-T cell exhaustion and impaired metabolic fitness, limiting the proliferative capacity, cytotoxicity, and long-term persistence of the cells.^6^ Regarding extrinsic factors, three key challenges have been defined: (i) antigen heterogeneity, as solid tumors often lack uniform expression of a single target antigen, leading to incomplete eradication and relapse; (ii) immunosuppression within the tumor microenvironment (TME), which is frequently enriched with immunosuppressive cytokines, regulatory cells, and metabolic constraints such as hypoxia and nutrient depletion; and (iii) inefficient trafficking of effector cells into and within tumor tissue. Although the immunosuppressive TME, which itself can block efficient infiltration, and antigen heterogeneity remain predominant limitations, the inability of systemically infused T cells to efficiently home to tumor sites is a major bottleneck limiting the efficacy of adoptive cell transfer therapies and forms the focus of this review.^7^^,^^8^

Inefficient migration of systemically infused CAR cellular therapy is a particular issue in the treatment of solid tumors, where target cells are not as easily reachable as in hematologic malignancies. Several preclinical and clinical studies in solid tumors, such as breast and lung cancer, have identified limited CAR-T cell persistence and infiltration as a main driver of poor responses.^7^^,^^8^ For CAR-T cells to reach their targets in solid tumors, they need to extravasate from the circulation, penetrate dense stromal barriers, and infiltrate the tumor core. Here, immune exclusion is an important driver of limited treatment efficacy, as cells often accumulate at the tumor margins but fail to infiltrate the tumor core. Several factors can drive immune exclusion, including hypoxia, a dense extracellular matrix limiting access to the tumor core, and a mismatched chemokine–chemokine receptor expression between tumors and T cells.^9^

**Figure 1:**
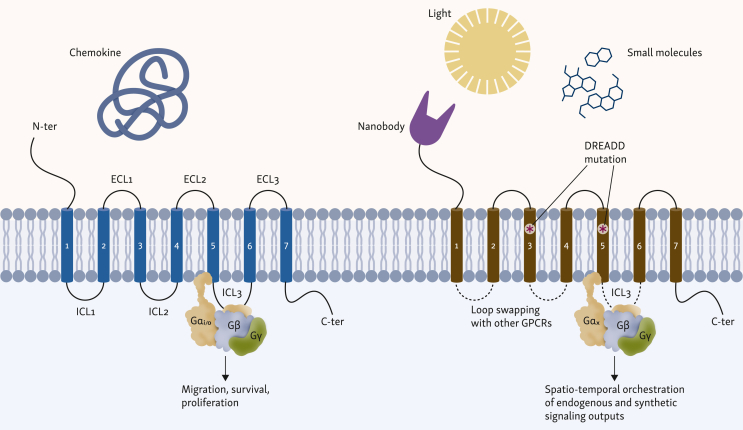
**Structural anatomy of natural versus engineered chemokine receptors and downstream signaling convergence.** Comparison of a canonical class A chemokine receptor (left) and a synthetically engineered variant (right). Natural receptors use the conserved 7-transmembrane (7TM) bundle to transmit environmental signals, whereas engineered receptors employ modular structural modifications to program specific behaviors. Regarding extracellular input, the N-terminus (N-ter) is fused with nanobodies or single-chain variable fragments to create programmable antigen-gated receptors (PAGERs), tethering activation to tumor antigen encounter. Mutations at the TM3/TM5 interface of the orthosteric pocket alter ligand specificity, generating designer receptors exclusively activated by designer drugs (DREADDs), which are insensitive to endogenous chemokines but responsive to bio-inert small molecules. Intracellular loops (ICL2 and ICL3) act as G protein-coupling interfaces. In OptoXR chimeras, chemokine receptor signaling loops (e.g. CXCR4) are grafted onto opsin scaffolds, converting photostimulation into specific migratory G protein cascades. Both receptor types converge on heterotrimeric G protein activation (Gαβγ) for downstream signaling. ECL, extracellular loop; TM3/TM5, third and fith transmembrane helices.

Cell trafficking is regulated by a complex network of molecular cues. GPCRs are a superfamily of membrane receptors that play a central role in this network and are essential mediators of cellular migration, making them attractive targets for engineering improved cellular immunotherapies. GPCRs constitute a vast protein family with around 800 membrane proteins. Currently, some 516 drugs are known to target these receptors, making GPCRs a field of vibrant drug research.^10^ GPCRs share a conserved structure motif of seven transmembrane helices connected by three intracellular and three extracellular loops.^11^ They can be activated by various stimuli, including light, ions, small molecules, and proteins, leading to complex downstream signaling and corresponding cellular responses. Remarkably, they all signal through the heterotrimeric G protein complex (Gαβγ) and arrestins (Figure 1).^12^ These receptors can be further grouped into four major families depending on the G alpha subunits used for signaling (G_s_, G_i/o_, G_q/11_, and G_12/13_).^13^ The combination of how the ligand stabilizes the GPCR, the homo- or heterodimerization of GPCRs, the environment of the cell, and allocated G proteins leads to a precise and context-dependent signaling output.

**Figure 2:**
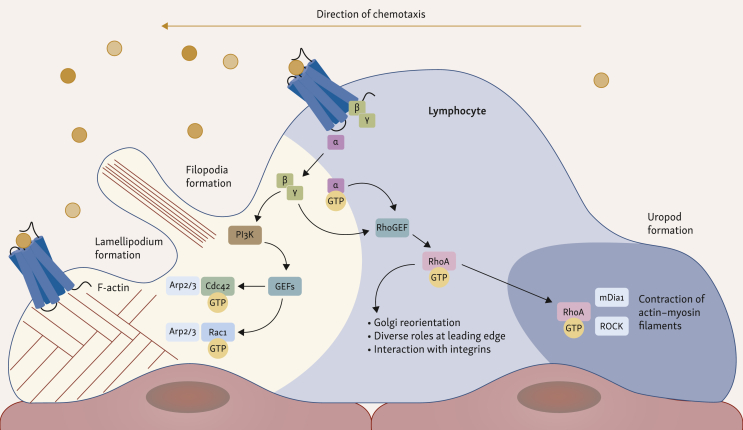
**GPCR-mediated signaling polarity during cell chemotaxis.** Simplified signaling cascade of GPCRs inducing chemotaxis and cell movements. Initially, GPCRs at the leading edge bind released chemokines, resulting in the dissociation of G proteins. The released Gβγ subunit locally activates GEFs via PI3K and PIP3, activating Rho GTPases. At the leading edge, Cdc42 promotes the formation of sensory, finger-like protrusions called filopodia, while Rac1 activation drives the formation of broad, sheet-like protrusions called lamellipodia. These GTPases mediate the polymerization of actin cytoskeleton by Arp2/3 complexes. At the trailing edge (uropod), the RhoA-ROCK signaling axis promotes the phosphorylation of myosin light chains, leading to contraction of actin-myosin filaments. The spatial segregation leads to Rac/Cdc42-driven protrusion at the front and RhoA-driven contraction at the rear. Arp2/3, actin-related protein 2/3 complex; Cdc42, cell division protein 42; GEF, guanine exchange factor; GPCR, G protein-coupled receptors; GTPase, guanosine triphosphatase; mDia1, mammalian Diaphenous-related formin 1; PI3K, phosphoinositide 3-kinase; PIP3, phosphatidylinositol 3,4,5-trisphosphate; Rac1, Ras-related C3 botulinum toxin substrate 1; ROCK, Rho-associated protein kinase.

**Figure 3:**
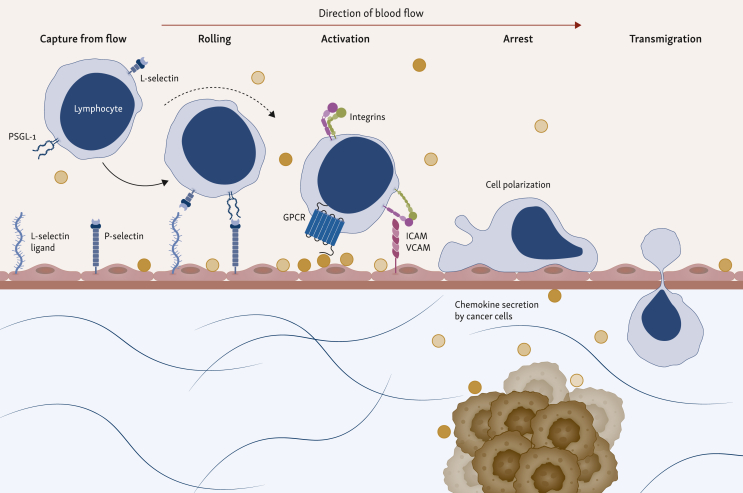
**The leukocyte adhesion cascade driving immune cell infiltration into the tumor microenvironment (TME).** The multistep process of leukocyte extravasation is initiated by the capture and rolling of leukocytes from the blood flow on the vascular endothelium. Low-affinity interactions mediate this between selectins and their carbohydrate ligands, namely P-selectin glycoprotein ligand-1 (PSGL-1) and L-selectin binding to endothelial P-selectin and L-selectin ligands. Rolling leukocytes bind chemokines secreted by cells of the TME displayed by proteoglycans on the endothelial surface. Binding activates leukocyte integrins by GPCR-transmitted signaling (inside-out signaling). This induces a high-affinity conformational change, leading to firm arrest. This arrest is stabilized by the binding of activated integrins like lymphocyte function-associated antigen 1 (LFA-1) to its endothelial ligand, intercellular adhesion molecule 1 (ICAM-1) and very late antigen-4 (VLA-4) to its ligand vascular cell adhesion molecule-1 (VCAM-1). Once firmly arrested, leukocytes begin transendothelial migration by squeezing through endothelial cell junctions and further navigating to the tumor. GPCR, G protein-coupled receptors.

One of the main GPCR families is that of chemokine receptors, which are key mediators of immune cell trafficking and positioning in the body. These receptors are classified into four structural groups based on the arrangement of conserved cysteine residues: C, CC, CXC, and CX3C. Within the chemokine–chemokine receptor system there is extensive redundancy: many chemokines can bind multiple receptors and many receptors recognize several chemokines, resulting in a complex, overlapping network.^14^ Some of the main downstream signaling pathways engaged by GPCRs include the PI3K/Akt, PLC and MAPK/ERK pathways. Collectively, these pathways orchestrate cytoskeletal reorganization, adhesion molecule regulation, and directional sensing, which are all essential for chemotaxis (Figure 2).^15^ Effective immune cell trafficking is a multistep process that involves adhesion and rolling along the vascular endothelium, transendothelial migration, and infiltration into tissues (Figure 3). These steps are all driven by the expression of chemokine receptors.^15^^,^^16^

Under healthy conditions, chemokine receptors coordinate the migration of immune cells to sites of infection or inflammation, thereby maintaining immune surveillance and tissue homeostasis. However, in cancer, the chemokine receptor system is frequently dysregulated. Tumor, stromal, and immune cells within the TME often up-regulate chemokine production, which can attract immunosuppressive cells such as regulatory T cells and myeloid-derived suppressor cells. Their recruitment remodels the microenvironment toward immune suppression, supporting tumor growth, metastasis, and treatment resistance. In contrast, important effector cells, such as T or NK cells, often lack expression of chemokine receptors that match the chemokine gradients present in the TME. This creates a barrier to effective migration to the TME and impairs immune-mediated tumor clearance. To overcome this frontier, adoptive immune cells can be engineered to express additional chemokine receptors to drive selective recruitment to the tumor site.^14^^,^^17^

This review discusses current and emerging approaches for GPCR engineering to enhance the migratory capacity and therapeutic impact of NK and T cells in cancer immunotherapy. Approaches using natural GPCRs will be discussed with a primary focus on chemokine receptors. Additionally, synthetic GPCR engineering will be discussed as a more recent strategy to modulate cellular function in a controlled manner.

## Natural GPCR engineering for improved migration

### Chemokine receptor engineering

A frequent challenge in adoptive cell therapy is the mismatch between chemokines secreted within the TME and the chemokine receptor repertoire expressed on effector immune cells. This discrepancy has prompted efforts to exploit chemokine–chemokine receptor axes present in tumors. In these approaches, immune effector cells are genetically engineered to express chemokine receptors required for migration toward tumor-derived chemokines. Most studies have focused on its application in T or NK cells, with more limited attempts in other cell types, which will be discussed later. The first proof-of-concept study by Kershaw et al. showed that engineering T cells with CXCR2 enhanced migration toward CXCL1-secreting human melanoma cell lines.^18^ Since then, numerous chemokine receptors have been evaluated in both T and NK cell platforms: CXCR1,^19^, ^20^, ^21^, ^22^, ^23^ CXCR2,^18^, ^19^, ^20^^,^^23^, ^24^, ^25^, ^26^, ^27^, ^28^, ^29^, ^30^, ^31^, ^32^, ^33^, ^34^, ^35^, ^36^, ^37^ CX3CR1,^38^ CXCR3,^39^^,^^40^ CXCR4,^41^, ^42^, ^43^, ^44^, ^45^, ^46^, ^47^, ^48^, ^49^, ^50^, ^51^, ^52^ CXCR5,^53^^,^^54^ CXCR6,^32^^,^^55^ CCR2,^56^, ^57^, ^58^, ^59^, ^60^, ^61^ CCR4,^60^, ^61^, ^62^, ^63^ CCR5,^64^ CCR6,^65^ CCR7,^48^^,^^66^, ^67^, ^68^ and CCR8.^69^ The respective ligands for these receptors are frequently overexpressed in the vicinity of various cancers. In contrast, expression of the corresponding chemokine receptors on effector cells is often down-regulated or absent, particularly following *ex vivo* expansion.^24^^,^^26^^,^^32^^,^^50^^,^^51^^,^^56^ Table 1 provides an exhaustive overview of published studies employing chemokine receptor engineering in T and NK cells.

**Table 1:** Chemokine receptor engineering to improve T or natural killer (NK) cell migration. Chemokine receptors are classified by type and studies are ordered chronologically from the earliest to newest study. Key findings have been highlighted in bold.

Chemokine receptor	Ref	Effector cell type	Method of engineering	Cancer type	Migratory effects and *in vivo* results
CXCR1	^19^	Anti-A20-28z+ chimeric antigen receptor (CAR)-T cells	Retroviral transduction	Ovarian, breast and pancreatic	Improved migration *in vitro*^19^, ^20^, ^21^, ^22^, ^23^ Improved migration *in vivo*^20^^,^^22^^,^^23^ **Enhanced antitumor efficacy *in vivo* in xenograft models**^20^^,^^22^
	^20^	Anti-CD70 CAR-T cells	Retroviral transduction	Glioblastoma, ovarian and pancreatic	
	^21^	Tumor-infiltrating lymphocytes (TILs)	mRNA electroporation	Melanoma	
	^22^	Anti-NKG2D CAR-NK cells	mRNA electroporation	Head and neck, and ovarian cancer	
	^23^	Anti-mesothelin (MSLN)-CAR-NK cells	Lentiviral transduction	Pancreatic cancer	
CXCR2	^18^	T cells	Retroviral transduction	None	Improved migration *in vitro*^18^, ^19^, ^20^^,^^23^, ^24^, ^25^, ^26^, ^27^, ^28^, ^29^, ^30^, ^31^, ^32^, ^33^, ^34^, ^35^, ^36^, ^37^ Improved migration *in vivo*^19^^,^^20^^,^^23^, ^24^, ^25^^,^^27^, ^28^, ^29^, ^30^, ^31^, ^32^, ^33^, ^34^, ^35^^,^^37^ **Enhanced antitumor efficacy *in vivo* in xenograft**^19^^,^^20^^,^^27^^,^^28^^,^^30^, ^31^, ^32^^,^^34^^,^^35^^,^^37^**and syngeneic**^24^^,^^29^**models**
	^24^	Transgenic pmeI-1 T cells	Retroviral transduction	Melanoma	
	^26^	Tumor ascites lymphocytes (TALs)	Lentiviral transduction	Ovarian cancer	
	^25^	TILs	Lentiviral transduction	Melanoma	
	^19^	Anti-A20-28z+ CAR-T cells	Retroviral transduction	Ovarian, breast and pancreatic	
	^20^	Anti-CD70 CAR-T cells	Retroviral transduction	Glioblastoma, ovarian and pancreatic	
	^27^	Anti-GPC3 CAR-T cells	Retroviral transduction	Hepatocellular carcinoma	
	^28^	iPSC-derived CAR-T cells	Gene-engineering at iPSC level	Breast and ovarian	
	^29^	Anti-claudin (CLDN)18.2 CAR-T cells	Lentiviral transduction	Pancreatic ductal adenocarcinoma (PDAC)	
	^30^	Anti-B7-H3 CAR-T cells	Retroviral transduction	Rhabdomyosarcoma and osteosarcoma	
	^31^	Canine anti-B7-H3 CAR-T cells	Retroviral transduction	Osteosarcoma	
	^32^	Anti-B7-H3 CAR-T cells	Lentiviral transduction	Osteosarcoma	
	^33^	Induced pluripotent stem cell (iPSC)-derived anti-HER2 CAR-T cells	Gene-engineering at iPSC level	Ovarian cancer	
	^34^	Anti-GPC2 CAR-T cells	Retroviral transduction	Neuroblastoma	
	^35^	NK-92 cells	Unknown	Osteosarcoma	
	^36^	NK cells	Retroviral transduction	Renal cell carcinoma	
	^37^	NK-92 cells	CRISPR-Cas9 gene editing	Colon cancer	
	^23^	Anti-MSLN-CAR-NK cells	Lentiviral transduction	Pancreatic cancer	
CXCR3	^39^	NK-92-αFR-CAR	Lentiviral transduction	Ovarian cancer	Improved migration *in vitro* and *in vivo*^39^^,^^40^ **Enhanced antitumor efficacy *in vivo* in xenograft models**^39^^,^^40^
	^40^	Anti-B7-H3 CAR-T cells	Lentiviral transduction	Diffuse intrinsic pontine glioma (DIPG)	
CX3CR1	^38^	T cells	Retroviral transduction	Colon cancer	Improved migration *in vitro* and *in vivo* **Enhanced antitumor efficacy *in vivo* in xenograft model**
CXCR4	^41^	Anti-c-kit CAR-T cells	Retroviral transduction	Hematopoietic stem cell transplantation (HSCT)	Improved migration *in vitro*^43^, ^44^, ^45^, ^46^, ^47^, ^48^, ^49^, ^50^ Improved migration *in vivo*^41^, ^42^, ^43^, ^44^, ^45^, ^46^^,^^48^^,^^49^^,^^51^^,^^52^ **Enhanced antitumor efficacy *in vivo* in xenograft**^42^^,^^43^^,^^45^^,^^48^^,^^49^^,^^51^^,^^52^**and immunocompetent allograft model**^44^
	^42^	Anti-CD25 CAR-T cells	Lentiviral transduction	Acute myeloid leukemia (AML)	
	^43^	Anti-CD33-CAR cytokine-induced killer cells (CIKs)	Sleeping beauty transposon system	AML	
	^44^	Anti-CLDN18.2 CAR-T cells	Retroviral transduction	PDAC	
	^45^	Anti-EGFRvIII-CAR-NK cells	Lentiviral transduction	Glioblastoma	
	^46^	NK cells	mRNA transfection	Bone marrow homing	
	^47^	Anti-CD19-CAR-NK cells	Lentiviral transduction	AML	
	^48^	NK-92 cells	Unknown	Colorectal cancer	
	^49^	Anti-BCMA CAR-NK cells	mRNA electroporation	Multiple myeloma	
	^50^	Anti-BCMA CAR-NK cells	Retroviral transduction	Multiple myeloma	
	^51^	CD7 KO anti-CD7-CAR-iNK cells	Genetic engineering at hPSC level using PiggyBac transposon system	T-cell acute lymphoblastic leukemia (T-ALL)	
	^52^	Anti-CD19-CAR-iNKP cells	Genetic engineering at hPSC level using PiggyBac transposon system	B-cell acute lymphoblastic leukemia (B-ALL)	
CXCR5	^53^	Anti-EGFR CAR-T cells	Lentiviral transduction	Non-small-cell lung cancer (NSCLC)	Improved migration *in vitro*^53^^,^^54^ Improved migration *in vivo*^53^ **Enhanced antitumor efficacy *in vivo* in xenograft model**^53^
	^54^	1G4 TCR-T cells	Lentiviral transduction	None	
CXCR6	^55^	OT-1 T cells, anti-EpCAM-CAR and anti-MSLN-CAR-T cells	Retroviral transduction	Pancreatic and ovarian cancer	Improved migration *in vitro*^32^^,^^55^ Improved migration *in vivo*^32^^,^^55^ **Enhanced antitumor efficacy *in vivo* in xenograft**^32^^,^^55^**and syngeneic**^55^**models**
	^32^	Anti-B7-H3 CAR-T cells	Lentiviral transduction	Osteosarcoma	
CCR2	^56^	Anti-GD2-CAR-T cells	Retroviral transduction	Neuroblastoma	Improved migration *in vitro*^56^, ^57^, ^58^, ^59^, ^60^, ^61^ Improved migration *in vivo* in xenograft^56^, ^57^, ^58^^,^^60^ and transgenic model^59^ **Enhanced antitumor efficacy *in vivo* in xenograft model**^56^, ^57^, ^58^^,^^60^**and transgenic model**^59^
	^57^	Anti-MSLN-CAR-T cells	Lentiviral transduction	Malignant pleural mesothelioma	
	^58^	WT1-specific TCR-T cells	Retroviral transduction	Lung cancer	
	^59^	SV40Tag-specific TCR-T cells	Retroviral transduction	Prostate cancer	
	^60^	Anti-MSLN-CAR-T cells	Lentiviral transduction	NSCLC	
	^61^	NK cells	Retroviral transduction	None	
CCR4	^62^	Anti-CD30 CAR-T cells	Retroviral transduction	Hodgkin lymphoma	Improved migration *in vitro*^60^, ^61^, ^62^, ^63^ Improved migration *in vivo* in xenograft^62^ and syngeneic model^63^ **Enhanced antitumor efficacy *in vivo* in xenograft**^62^**and syngeneic model**^63^
	^63^	T cells	Retroviral transduction	Pancreatic cancer	
	^60^	Anti-MSLN-CAR-T cells	Lentiviral transduction	NSCLC	
	^61^	Primary NK cells	Retroviral transduction	None	
CCR5	^64^	NK cells	Lentiviral transduction	Colon cancer, cervical cancer, breast cancer, chronic myelogenous leukemia	Improved migration *in vitro* and *in vivo* **Enhanced antitumor efficacy and prolonged survival *in vivo* in xenograft colon cancer model**
CCR6	^65^	Anti-EGFR CAR-T cells	Lentiviral transduction	Lung cancer	Improved migration *in vitro* Improved migration and **enhanced antitumor efficacy *in vivo* in xenograft model**
CCR7	^66^	NK cells	Trogocytosis	Lymph node homing	Improved migration *in vitro*^48^^,^^66^, ^67^, ^68^ Improved homing *in vivo*^48^^,^^66^^,^^68^ **Enhanced antitumor efficacy and prolonged survival *in vivo* in xenograft model**^48^^,^^68^
	^67^	NK cells	mRNA electroporation	Lymphoma	
	^48^	NK-92 cells	Lentiviral transduction	Colorectal cancer	
	^68^	Anti-CD19 CAR-aNK cells	DNA electroporation	Lymphoma	
CCR8	^69^	OT-1 T cells Anti-EpCAM CAR-T cells Anti-MSLN CAR-T cells	Retroviral transduction	Pancreatic cancer	Improved migration *in vitro* Improved migration *in vivo* in syngeneic and xenograft model **Enhanced antitumor efficacy *in vivo* in syngeneic and xenograft model**

BCMA, B-cell maturation antigen; CAR, chimeric antigen receptor; CD, clusters of differentiation; EGFR, epidermal growth factor; EpCAM, epithelial cell adhesion molecule; TCR, T-cell receptor.

Although these studies have identified chemokine receptor engineering as a promising approach, current studies are limited in their scope and impact by available methods. Most rely on *in vitro* transwell assays, which enable controlled investigation of chemotaxis, but lack the spatial and structural complexity of *in vivo* settings. *Ex vivo* models, such as organoids or organotypic slice cultures, offer greater physiological relevance and allow visualization of migration through fluorescent or confocal microscopy.^23^^,^^27^^,^^29^^,^^51^^,^^52^^,^^55^ Lastly, bioluminescence imaging and intravital two-photon microscopy enable dynamic tracking of CAR-T cell behavior *in vivo*.^24^^,^^32^^,^^39^^,^^55^^,^^56^^,^^59^^,^^60^ Although each of these methods provides useful information, they all come with their limitations and should be used in conjunction to obtain the best resolution.

#### Impact of chemokine receptor engineering on migration, tumor infiltration, and persistence

Genetic modification of T or NK cells to express chemokine receptors has consistently improved migration toward recombinant chemokine gradients and tumor-derived chemokine signals. As outlined in Table 1, when combined with a CAR, chemokine receptor expression enhances tumor homing and infiltration, improving antitumor responses and survival in most syngeneic or xenograft murine cancer models. Although increased migration is the primary benefit, chemokine receptor engineering has also been associated with additional effects, including enhanced cell proliferation, metabolic fitness, and remodeling of the TME.^30^^,^^31^^,^^34^^,^^41^^,^^44^^,^^56^ Some of these effects may result from preferential interactions with other cells in the TME, such as myeloid or dendritic cells (DCs).^70^

Among the chemokine receptors studied, CXCR2 has been most frequently investigated for its ability to enhance the trafficking of T or NK cells. It generally outperforms CXCR1, likely due to its broader chemokine ligand recognition.^19^^,^^20^^,^^23^ CXCR2-engineered T cells demonstrate enhanced tumor infiltration and persist at comparable levels to non-engineered T cells in the peripheral blood. This suggests that improved migration may also contribute to augmented *in situ* expansion upon tumor and CAR engagement.^20^^,^^24^^,^^27^^,^^32^ Methodologically, this could be validated by methods such as immunohistochemistry and bioluminescence imaging.^24^^,^^25^^,^^29^^,^^32^^,^^56^^,^^60^^,^^62^ Improved expansion has also been reported for CCR2- and CXCR4-expressing CAR-T cells.^41^^,^^56^

A gain-of-function mutant of CXCR4, the so-called CXCR4^R334X^ variant, has been explored for its ability to mediate migration. This mutant leads to increased chemotactic responsiveness toward its ligand SDF-1α following increased signal transduction, reduced receptor desensitization, and impaired receptor internalization.^43^^,^^46^^,^^50^ Although CXCR4^R334X^ shows superior migration *in vitro* and *in vivo* relative to wild-type CXCR4,^43^^,^^46^^,^^49^^,^^50^ one study reported reduced antitumor activity when using this mutant in the context of acute myeloid leukemia. Such an opposing effect was hypothesized to be linked to immunological synapse instability, underscoring the complexity of balancing trafficking with functionality.^43^

Certain chemokine ligands, such as CX3CL1 (for CX3CR1) and CXCL16 (for CXCR6), exist in both soluble and membrane-bound forms. In their membrane-bound form, these chemokines act as adhesion receptors expressed by activated endothelial cells, facilitating the recruitment of T cells from the circulation to the tumor site.^38^^,^^55^ Additionally, increased expression of the adhesion molecule VLA-4 has been observed on CXCR6-expressing T cells *in vivo*. This finding indicates that the CXCL16-CXCR6 axis is not only important for adhesion to the endothelium, but also for transendothelial migration.^55^ In a mouse osteosarcoma xenograft model, a comparative analysis of CXCR2 and CXCR6 revealed faster migratory kinetics and earlier tumor localization for CXCR2. CXCR6, in contrast, migrated more slowly but ultimately achieved greater tumor accumulation and persistence, independent of CAR engagement.^32^

#### Impact of chemokine receptor engineering on effector function and cytotoxicity

Chemokine receptor expression typically does not markedly alter overall effector function, subset distribution, or exhaustion profiles in T and NK cells.^30^^,^^34^ For CXCR2, IFN-γ and interleukin (IL)-2 secretion are generally comparable with controls in most studies,^19^^,^^24^^,^^27^^,^^32^^,^^65^ although some have reported increased cytokine release upon ligand or target engagement.^30^^,^^31^ Most studies find that cytotoxic functionality remains largely unchanged after chemokine receptor engineering,^19^^,^^20^^,^^25^^,^^27^^,^^28^ although enhanced killing has been reported in 3-dimensional spheroid and organoid models or under migration-restricting conditions for CXCR2.^27^ CXCR4-expressing T cells have shown an increase in cytokine release, proliferation, and cytotoxicity when cocultured with tumor cells in the presence of their ligand SDF-1α,^43^ although this effect was not reproduced in a 2-dimensional pancreatic ductal adenocarcinoma culture system.^44^ CXCR4-engineered CAR-NK cells have exhibited enhanced cytolytic activity compared with conventional CAR-NK cells. This is linked to improved ERK/AKT signaling, stabilization of CAR surface expression and the resulting immune synapse, and increased ZAP-70 recruitment. These changes increase sensitivity to low-antigen tumor cells even without SDF-1α stimulation.^50^ *In vitro*, CXCR6-CAR-T cells have been reported to exhibit improved cytolytic function due to increased adhesion to transmembrane CXCL16 on tumor cells.^55^ For CCR2, results have been mixed. Several studies reported no impact on cytotoxicity,^56^^,^^58^, ^59^, ^60^ whereas another observed augmented killing in coculture systems, especially in the presence of the CCR2 ligand CCL1.^57^ These seemingly opposing effects are difficult to interpret without true comparative evidence and may be due to differences in experimental systems, cell types, phenotypes, and assay conditions. This variability underpins the need for careful assessment of any newly designed product for changes in functionality.

Interestingly, CXCR2-expressing T cells exhibit a unique transcriptomic profile, characterized by metabolic changes such as increased spare respiratory capacity, enhanced glycolytic potential upon IL-8 stimulation, and elevated mitochondrial abundance even in the absence of IL-8. These changes could be attributed to activation of the PI3K/AKT pathway and downstream mTORC-1 signaling.^30^^,^^31^^,^^34^ Such metabolic advantages can support CXCR2-expressing cells in their effector functions, which is particularly beneficial in the nutrient-poor and hypoxic TME.^71^

#### Effects of chemokine receptor engineering on the tumor microenvironment

Studies utilizing immunocompetent mouse models have revealed that chemokine receptor augmentation can also reshape the suppressive TME. CXCR2-engineered CAR-T cells have been found to reduce the number of myeloid-derived suppressor cells (MDSCs) and CXCR2+ macrophages within tumors, thereby alleviating local immunosuppression.^29^^,^^34^ Similarly, CXCR4-augmented CAR-T cells can suppress MDSC recruitment through modulation of the STAT3/NF-kB/SDF-1α signaling axis.^44^ Receptors such as CXCR1 and CXCR2 may also serve to siphon their ligand IL-8, inhibiting its effects on angiogenesis and the recruitment of immunosuppressive cells.^20^ In an immunocompetent Panc02-OVA model, CCR4-engineered T cells demonstrate increased interactions with DCs. These interactions are facilitated by strengthened LFA-1-ICAM-1 binding, supporting sustained T-cell activity and proliferation in solid tumors.^63^ This effect is partly mediated by the co-expression of the CCR4 ligand CCL22 by immune cells such as DCs in several cancer types, including Hodgkin lymphoma (HL) and pancreatic cancer.^62^^,^^63^

#### Translation of chemokine receptor engineering to clinical trials

Despite the numerous preclinical studies conducted on enhancing cellular therapy through chemokine receptor engineering, their translation into clinical trials has been limited. Only engineered T cells have entered clinical testing, and most trials are in early phases. Table 2 provides an overview of the clinical trials that have been carried out or are currently ongoing. To date, only a single phase I/II trial (NCT01740557) has been completed, although detailed results regarding efficacy have not yet been published. The only other trial that has released some preliminary data is a phase I trial (NCT03602157) in relapsed/refractory CD30+ HL and cutaneous T-cell lymphoma (CTCL) that was initiated following promising preclinical results.^62^ Treatment was well-tolerated with no dose-limiting toxicities. Out of eight HL patients, six were complete and two were partial responders, with durable remissions including one ongoing at 2.5 years. Activity in CTCL patients was more limited, with one patient achieving stable disease as the best response and another experiencing progressive disease. Compared with CD30.CAR-Ts, CCR4.CD30.CAR-Ts showed enhanced tumor trafficking, supporting CCR4’s role in improving homing and antitumor activity.^72^ A second phase Ib/II clinical trial is ongoing in HL, with first results expected in late 2026 (NCT06090864).

**Table 2:** Clinical trials conducted or ongoing using chemokine receptor-engineering for adoptive T-cell therapies for cancer treatment

Chemokine receptor	ACT type	Treatment regimen	Phase	Cancer	Status	Trial number
CCR4	Anti-CD30 CAR-T cells with or without additional CCR4 expression	Lymphodepletion followed by 1-2 CAR-T cell administrations i.v.	I	Relapsed/refractory CD30+ Hodgkin (HL) and cutaneous T cell lymphoma (CTCL)	Recruiting, preliminary results published	NCT03602157
	CCR4 modified anti-CD30 CAR-T cells	Lymphodepletion followed by 1-2 CAR-T cell administrations i.v.	Ib/II	Relapsed/refractory CD30+ Hodgkin lymphoma (HL)	Recruiting	NCT06090864
CXCR2	CXCR2 and NGFR transduced TILs	Lymphodepletion followed by 1 i.v. TIL administration and subsequent high-dose aldesleukin i.v.	I/II	Stage III or metastatic melanoma	Completed, limited results posted	NCT01740557
	CXCR2 modified CD70 CAR-T cells	Chemoradiation followed by 1 CAR-T cell administration i.v.	I	CD70+ adult glioblastoma	Recruiting	NCT05353530
	CXCR2 modified CD70 CAR-T cells	Chemoradiation followed by lymphodepletion and 1 CAR-T cell administration i.v.	I	CD70+ pediatric high-grade glioma	Recruiting	NCT06946680
CXCR4	CXCR4-modified anti-BCMA CAR-T cells	i.v. administration CAR-T cells	I	Relapsed/refractory multiple myeloma	Recruiting	NCT04727008
	CXCR4 modified anti-CD19 CAR-T cells	i.v. administration CAR-T cells	I	Relapsed/refractory CD19+ B-cell malignancies	Unknown	NCT04684472
CXCR5	CXCR5-modified anti-EGFR CAR-T cells	i.v. administration CAR-T cells	I	Advanced non-small-cell lung cancer	Recruiting	NCT05060796
	CXCR5-modified anti-EGFR CAR-T cells	i.v. administration CAR-T cells	I	Advanced non-small-cell lung cancer	Unknown	NCT04153799

BCMA, B-cell maturation antigen; CAR, chimeric antigen receptor; CD, clusters of differentiation; EGFR, epidermal growth factor; i.v., intravenous; NGFR, nerve growth factor receptor.

#### Beyond T and NK cells

Although most chemokine receptor engineering efforts have focused on T and NK cells, other cell types also contribute to productive antitumor responses. DCs have a central and well-established role in orchestrating immunity, and mesenchymal stem cells (MSCs) are increasingly investigated as therapeutic vehicles in cancer. So far, exploitation of chemokine–chemokine receptor axes has barely been studied in these cell types, although such approaches warrant further research.

DCs play a crucial role in initiating antitumor immune responses by capturing and presenting tumor-associated antigens to T cells in lymph nodes.^73^ DC-based vaccines have been widely used to elicit T cell responses by loading DCs with relevant tumor-associated antigens.^74^ The efficacy of these therapies depends on efficient DC migration to tumor-draining lymph nodes (dLN). CCR7 is the main driver of DC migration from the tumor site to dLNs. Its expression can be suppressed by tumor cells, impeding their homing and permitting immune escape.^75^ Several studies have therefore engineered DCs to overexpress CCR7, resulting in improved lymph node homing and a subsequent enhanced efficacy of DC-based vaccines.^76^, ^77^, ^78^ Given the downfall of DC approaches in recent years, such avenues remain to be further exploited.

In addition to immune cells, certain stromal cell types are also being explored for chemokine receptor engineering in cancer therapy. Among them, MSCs are particularly attractive because of their strong tropism toward tumors. Although they can promote tumor growth, metastasis, and immune evasion, they are also being explored as vehicles for delivering targeted cancer therapies, exploiting their innate homing abilities.^79^ In this context, engineering MSCs to overexpress tumor-relevant chemokine receptors can further enhance their migration and delivery of therapeutics to tumor sites. MSCs have, for example, been engineered to overexpress CXCR4, which is often down-regulated upon *ex vivo* expansion.^80^ CXCR4 overexpression augmented migration *in vitro*,^81^^,^^82^ as well as *in vivo* in a triple-negative breast cancer xenograft model.^81^ CXCR4-engineered MSCs could also reduce tumor burden in a model of colitis-associated cancer. These findings indicate that MSCs are not only of interest as drug carriers, but also possess intrinsic antitumor properties and hold promise as a cellular therapy. MSCs have also been engineered to express CCR2, CCR7, CCR5, and CXCR6, though for applications other than cancer, such as for the treatment of acute ischemic stroke, graft-versus-host disease and retinophathies.^83^, ^84^, ^85^ It is tempting to speculate that similarly modified MSCs might be usable in malignancies with compatible chemokine profiles.

## Synthetic GPCR engineering: overcoming the limits of the tumor milieu

Natural chemokine receptor engineering, although effective for improving homing, is often limited by the heterogeneous and unpredictable chemokine landscape of the TME. To achieve trafficking that is independent of these endogenous signals, synthetic GPCR engineering approaches have been developed (Figure 1).

### Chemogenetic modulation of cell functions

Chemogenetics describes a process in which chemically engineered receptors and specific exogenous ligands are used to control cellular activities. In the context of cellular therapy, this approach offers an orthogonal solution for directed cell migration.

To analyze specific downstream effects of Gi-coupled receptors, a new family of receptors was engineered to be activated solely by a synthetic ligand (RASSL).^86^ This mutated human κ opioid receptor was designed to bind the synthetic agonist spiradoline, enabling orthogonal signaling. Next-generation designer receptors exclusively activated by a designer drug (DREADDs) decreased basal activation of RASSLs (Figure 1). These receptors utilized a family of mutated muscarinic acetylcholine receptors, which retained canonical G protein-coupling specificities after binding the synthetic ligand clozapine-N-oxide (CNO). This approach aimed to analyze different types of GPCRs in the parasympathetic nervous system.^87^ DREADDs were further explored in neuroscience to facilitate specific neuron firing using the human M4 muscarinic DREADD receptor^88^ *in vitro* and *in vivo*.^89^^,^^90^

Beyond neurology, the application of DREADDs to immune cell trafficking has shown that synthetic ligands can trigger the leukocyte adhesion cascade, as described in Figure 3. Gαi DREADD expressing cells showed robust chemotaxis to CNO in a transwell assay. Directed migration of transduced T cells was also shown *in vivo* using CNO-releasing microspheres.^91^ Although the field of DREADDs moved into neuroscience,^88^ a different GPCR-based platform termed programmable antigen-gated G protein-coupled engineered receptors (PAGERs)^92^ was developed. In this platform, different DREADD scaffolds were fused with a conditional auto-inhibitory domain and a nanobody. Upon binding of the nanobody to its target, the auto-inhibition is relieved, allowing small-molecule drugs to activate the receptor. Using endogenous signaling of Gi-coupled receptors, an anti-mCherry PAGER_GI_ in combination with the synthetic drug deschloroclozapine induced migration of primary human T cells along an mCherry gradient. Overall, although their primary focus lies in biomedical brain research, these chemically engineered receptors have shown potential applications in lymphocyte engineering, as demonstrated by the use of DREADDs for *in vivo* migration.^93^

### Optogenetic modulation of cell functions

In addition to chemogenetics, optogenetics offers a complementary approach to modulate cell migration with high spatial and temporal precision, which is necessary to overcome the physical barriers of the TME.

Optogenetics controls cellular activities through light, typically using so-called photoreceptors. Once activated by light, energy of the chromophore-based photoreceptor is transferred to a different module, where light can be used to control endogenous proteins, engineered fusion proteins, or chimeras of opsins. Photoreceptors can control neuronal firing, subcellular localization of protein activity, or alter protein influence.^94^ Compared with small molecules, light offers precise control of spatial and temporal signal, easy modulation, and an inexpensive and highly specific substrate, which has been exploited to analyze cellular activation pathways.^95^

The following paragraphs outline optogenetic approaches to influence cell migration and polarization using GPCRs as the backbone molecule and their heterotrimeric G proteins as signaling molecules. Direct control of GPCR pathways can be achieved by opsins, which are naturally occurring light-activated GPCRs found in animals' visual and non-visual systems. Upon light stimulation, retinal cofactors isomerize and induce a conformational change that initiates G protein signaling-cascades. For instance, blue opsin recruits endogenous Gi/o-coupled downstream pathways, generating local phosphatidylinositol 3,4,5-trisphosphate (PIP3) gradients and triggering lamellipodia formation, thereby driving the directional migration of the murine macrophage-like cell line RAW264.7 toward the light stimulus.^96^

An alternative approach to inducing migration is to act directly on GPCR-associated G proteins. O’Neill and Gautam developed two optogenetic constructs that target endogenous G protein signaling in macrophages. Upon exposure to blue light, signaling is dampened by recruiting specific inhibitors to the membrane, including RGS4, which accelerates Gα deactivation, and GRK2ct, which sequesters Gβγ subunits. This spatially restricted light exposure enables control over the intracellular location in which signaling is inhibited. This effect may direct lamellipodial protrusions and guide cell migration directionally.^97^ Similarly, guanine nucleotide dissociation inhibitors (GDIs) based on the G protein regularity motif of activators of G protein signaling 3 (AGS3) were fused with the blue light-responsive CRY2 photoreceptor. This OptoGDI was recruited to the cell surface upon blue light stimulation, stabilizing GαGDP subunits and releasing free Gβγ subunits independently of GPCR or Gα activation. This led to localized generation of PIP3 and created a signaling gradient inside the cell, resulting in migration of macrophage RAW 264.7 cells.^98^ This converging evidence suggests that the Gβγ subunits are the driving force of optogenetic migration control.

A third method to induce cell modulation replaces intracellular loops of a GPCR of interest with those of a light-sensing GPCR, resulting in chimeric OptoXRs. Pioneering work generated opto-β2AR and opto-α1AR chimeras for optical control of mammal behavior.^99^ This strategy was then used to create photoactivatable chemokine receptors for improved infiltration of adoptively transferred cells. In detail, a photoactivatable chimera of a light-sensitive rhodopsin and the intracellular loops and C-terminus of human CXCR4 was created. This PA-CXCR4 construct induced cell polarization, canonical Gαi activation, and migration of transduced T cells to B16-OVA melanoma tumors when illuminated by light of 505 nm wavelength, resulting in reduced tumor growth compared with control mice.^100^ To date, this remains the only cancer-related translational approach. Additionally, this approach could be used to engineer Frizzled7 receptors into rhodopsin for the analysis of non-canonical Wnt signaling and the migration of prechordal plate cells *in vivo*, utilizing zebrafish embryos.^101^

Other optogenetic approaches, which do not rely on GPCRs to induce migration are also emerging. One such approach is the use of engineered receptor tyrosine kinases, which can also be optogenetically altered to facilitate cell migration.^102^ However, low tissue penetration, especially of shorter wavelength lights, limits the effectiveness *in vivo* due to poor delivery. One solution is the use of upconverting nanoparticles, which can emit blue light after near infrared radiation to achieve deeper tissue penetration.^103^

In summary, the field has taken essential steps to establish optogenetics for cell modulation. Although current applications have mainly focused on elucidating cell signal mechanisms, an interesting combination of chimeric optogenetic receptors could be possible following the work of Xu and colleagues.^101^

## Conclusion and outlook

Engineering of effector T and NK cells with a multitude of chemokine receptors has shown substantial potential to improve migration and antitumor efficacy in various malignancies. This approach enables more targeted recruitment of effector cells to the tumor site and can confer additional benefits such as increased cytotoxicity, TME remodeling, and increased persistence. Currently, repeated administrations at high cell numbers are often required to achieve successful therapeutic outcomes. This is in part due to inefficient tumor homing and the non-specific distribution of cells throughout the body.^4^ Directing CAR-T or CAR-NK cells to the tumor more efficiently could reduce the number of cells needed and lead to fewer on-target/off-tumor effects, reducing toxicity. Such a reduction in toxicity was reported in osteosarcoma and pancreatic cancer xenograft models when mice were treated with CXCR2 or CXCR6-expressing CAR-T cells compared with mice treated with unmodified CAR-T cells.^19^^,^^32^

However, this approach is not without risk. Off-tumor trafficking may occur when the targeted chemokines are also expressed in healthy tissues. Examples include the CCR2-CCL2 axis, which is involved in the recruitment of monocytes and T cells to inflamed tissues, and the CXCR4-CXCL12 axis, which controls retention of hematopoietic and immune cells within bone marrow and other CXCL12-rich organs.^17^^,^^104^ Additionally, certain chemokine receptors might redirect the engineered cells to immunosuppressive niches, potentially limiting their therapeutic efficacy instead of enhancing it. It is therefore important to carefully select appropriate chemokine receptor–ligand pairs or to implement further engineering strategies to overcome local immunosuppression.^69^

Although T and NK cells have been the primary focus of chemokine receptor engineering, limited work in other cell types, such as DCs and MDSCs, suggests that this strategy could have broader application. In this context, CAR macrophages have emerged as a promising immunotherapeutic platform, and it will be interesting to explore whether their antitumor activity can similarly be enhanced by targeted chemokine receptor engineering.

Despite these advances, the use of natural GPCRs such as chemokine receptors has some drawbacks. Most notably, their effectiveness depends on the specific chemokine milieu produced by the tumor, which is highly variable between and within tumor entities and can change over time. This context dependence limits the broad applicability of this approach. Synthetic GPCR engineering has the potential to overcome this limitation by enabling cells to migrate to an external stimulus of choice, making it applicable to any cancer type regardless of the chemokine environment present. However, this strategy is currently not without challenges and limitations, including complex engineering attempts and challenging delivery. These challenges are reflected by the limited number of publications exploiting these synthetic systems for targeted therapy. Nevertheless, both chemokine receptor and synthetic GPCR engineering approaches hold great promise for improving the precision and efficacy of cell-based cancer therapies. Further research will be necessary to fully realize the therapeutic potential of these approaches.
